# A novel care guide for personalised palliative care – a national initiative for improved quality of care

**DOI:** 10.1186/s12904-021-00874-4

**Published:** 2021-11-11

**Authors:** Dröfn Birgisdóttir, Anette Duarte, Anna Dahlman, Bengt Sallerfors, Birgit H. Rasmussen, Carl Johan Fürst

**Affiliations:** 1 Faculty of Medicine, Department of Clinical Sciences Lund, Oncology and Pathology, Institute for Palliative Care, Lund University, Scheeletorget 1, Hus 404B, 223 81 Lund, Sweden; 2 The Institute for Palliative Care, Lund University and Region Skåne, Lund, Sweden; 3 Faculty of Medicine, Department for Healthcare Sciences, Institute for Palliative Care, Lund University, Lund, Sweden

**Keywords:** Quality improvement, Clinical practice guidelines, Decision support, Palliative care, Dying, Clinical pathway, Patient-centred care, Personalised care, Early identification, Early Palliative Care

## Abstract

**Background:**

Even when palliative care is an integrated part of the healthcare system, the quality is still substandard for many patients and often initiated too late. There is a lack of structured guidelines for identifying and caring for patients; in particular for those with early palliative care needs. A care guide can act as a compass for best practice and support the care of patients throughout their palliative trajectory. Such a guide should both meet the needs of health care professionals and patients and families, facilitating discussion around end-of-life decision-making and enabling them to plan for the remaining time in life. The aim of this article is to describe the development and pilot testing of a novel Swedish palliative care guide.

**Methods:**

The Swedish Palliative Care Guide (S-PCG) was developed according to the Medical Research Council framework and based on national and international guidelines for good palliative care. An interdisciplinary national advisory committee of over 90 health care professionals together with patient, family and public representatives were engaged in the process. The feasibility was tested in three pilot studies in different care settings.

**Results:**

After extensive multi-unit and interprofessional testing and evaluation, the S-PCG contains three parts that can be used independently to identify, assess, address, follow up, and document the individual symptoms and care-needs throughout the whole palliative care trajectory. The S-PCG can provide a comprehensive overview and shared understanding of the patients’ needs and possibilities for ensuring optimal quality of life, the family included.

**Conclusions:**

Based on broad professional cooperation, patients and family participation and clinical testing, the S-PCG provides unique interprofessional guidance for assessment and holistic care of patients with palliative care needs, promotes support to the family, and when properly used supports high-quality personalised palliative care throughout the palliative trajectory. Future steps for the S-PCG, entails scientific evaluation of the clinical impact and effect of S-PCG in different care settings – including implementation, patient and family outcomes, and experiences of patient, family and personnel.

**Supplementary Information:**

The online version contains supplementary material available at 10.1186/s12904-021-00874-4.

## Background

For many patients with terminal illness, access to and quality of palliative care is substandard and random [1–3]. This leads to unnecessary suffering for patients and families left without adequate interventions and support. Evidence-based palliative care as well as patient involvement in decisions and the caring process are promoted by international [4–8] and Swedish national recommendations and the Swedish law [9, 10]. One of the major challenges for improved palliative care is the operationalization of such recommendations [11, 12]. It is well known that in spite of the general and legal aims for evidence-based care, it is a challenge to transform evidence-based guidelines, whether national, regional or local, into clinical practice [11, 13–16]. In order to improve the outcome of care for patients and families there is a need to change the behaviour among health care workers [17, 18]. The most common approach is education aimed at augmenting knowledge, attitudes and skills. Interventions that are most likely to attain behavioural change in health care often combines: restructuring of practice, altering of norms and attitudes (e.g. through education), together with external audits and feedback [19] and sensemaking [20].

Several tools have been developed to support the process of screening for palliative care needs and to guide the team to take necessary action [21–25]. The tools include overarching guidance for future care planning based on a number of prompts supporting a comprehensive assessment and care involving patient, family and team members. Clinical guidelines and pathways have also been designed to help health care professionals make relevant decisions and guide best-practice care [11, 19, 21, 26, 27]. One example from end-of-life care is the Liverpool Care Pathway (LCP) [28], which has been embraced as a useful guide for the care of the dying patient but also encountered strong critique [15, 29–32].

An unmet demand for early identification of palliative care needs is evident, but finding the patient with palliative care needs, and systematically assess and address such needs, is a challenge for professionals in most health care settings [33–38]. This calls for a systematic approach even for those working in specialized palliative care. The Swedish health care professionals working with palliative care at the end-of-life have called for a more supportive structured around care for patients earlier on in the palliative trajectory. A more proactive approach to palliative care is also encouraged by the World Health Organization [39], and several initiatives, including new development of clinical guides to promote care of the dying, have already moved in this direction [5, 25, 40–43]. Early integration of palliative care competency and early identification of patient needs have been shown to be effective in reducing suffering, increasing quality of life, and even prolonging survival [44–47].

With the ambition to meet the challenges of transforming knowledge into clinical palliative care practice we have developed a guide, named “The Swedish Palliative Care Guide” (S-PCG), to inform best practice and to meet the palliative care needs of patients and families throughout the palliative trajectory. The guide aims to provide support for a timely initiation of evidence-based personalised palliative care and is designed to meet palliative care needs on an individual basis. The guide should support that the quality of care is adequate for every adult patient and family with palliative care needs regardless of diagnosis or place of care (at home, or in a residential care home, hospice, or hospitals) and cover the whole palliative care trajectory. Throughout the development of the guide, it has been in the forefront to support integration of the principles of good palliative care into clinical practice rather than just provide strict instructions for implementation.

Our purpose in developing the S-PCG was to provide support to any given interdisciplinary team at a health care facility, helping them to provide the best possible personalised palliative care. The S-PCG aim is to help identify patients, assess palliative care needs, give decision support and help choose relevant care interventions, in order to enhance the greatest possible well-being of patients with limited time left in life. The aim of this article is to describe the comprehensive development process and the resulting “product” of the S-PCG.

## Method and process of development

### Study design

The work of compiling and testing the guide was carried out in 2013-2016. We used the Medical Research Council (MRC) framework to provide a robust structure for the process [48]. This article describes the phases of developing as well as feasibility and piloting. The study followed the ethical guidelines stated in the Declaration of Helsinki [49] and was performed in accordance to the Swedish laws and the local and national ethical review authority considerations concerning quality improvements and clinical audit within the health care.

### Developing

#### Reviewing the current standards for palliative care and defining the need for guidance

A steering committee oversaw the project, provided strategy and performed stakeholder analysis. A project group modelled the new care guide and led the testing of it. A national interdisciplinary advisory committee was established to review the content of the S-PCG. In order to cover the full range of the palliative care team and represent the different fields of health care, it included 95 health care professionals, researchers and others relevant for patients with palliative care needs. (Supplementary table A).

The S-PCG was designed based on current national and international evidence as described in regulatory documents issued by health care authorities, specifically the 2013 National Guidelines for Good Palliative Care at the End of Life [9], the 2012–2014 National Program for Palliative Care [50], together with quality indicators in the Swedish Palliative Registry and other relevant national indicators [51, 52]. The sections in the S-PCG on care for the dying person and care of the deceased person were inspired by the Liverpool Care Pathway (LCP) [28] and included the key principles and core elements from the 10/40 model set up by the International Collaborative for the Best Care of the Dying Person [53, 54]. The 10/40 model includes description of the ten principles together with the 40 core elements, used as quality indicators for good palliative and demonstrate good palliative care [54].

**Fig. 1:**
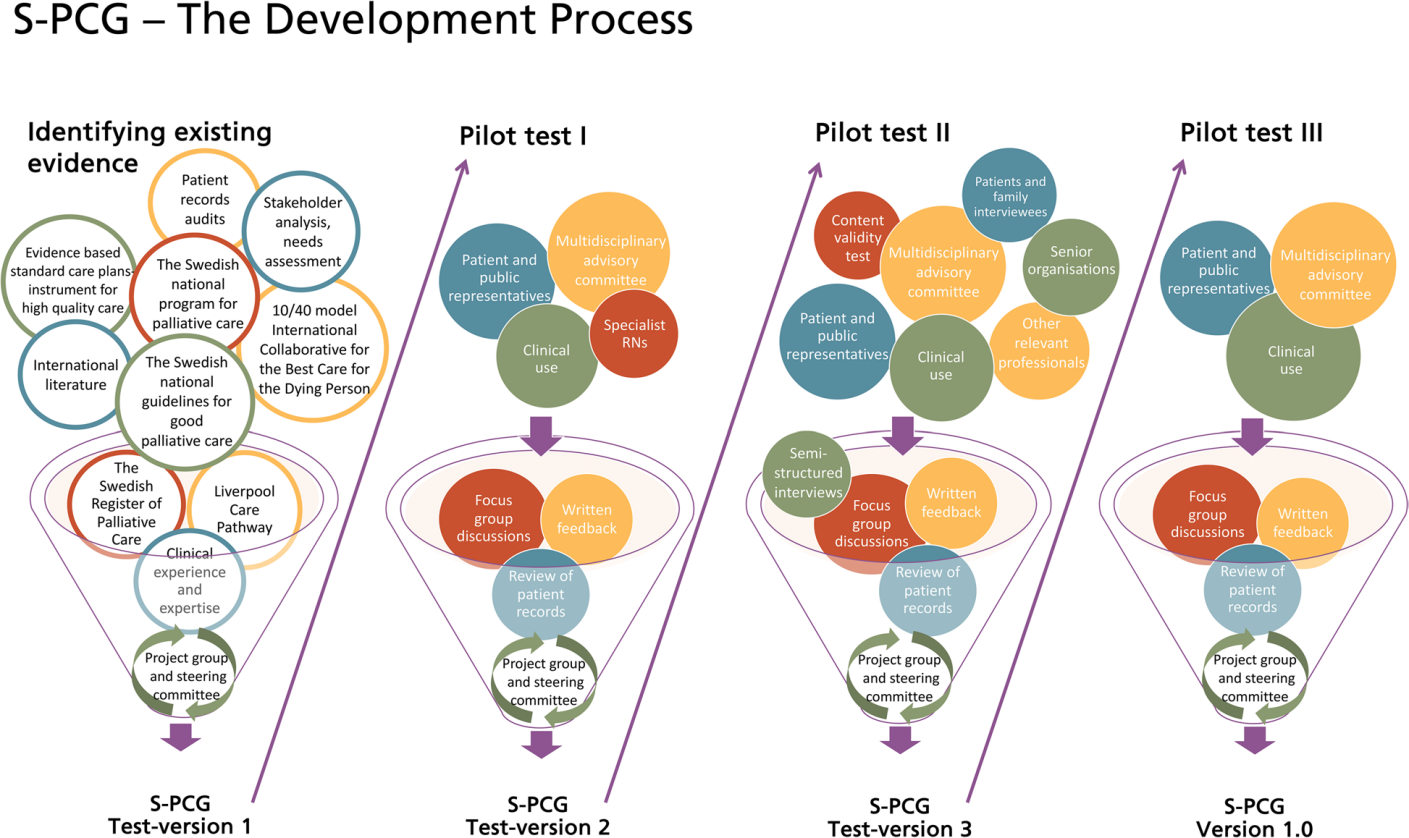
Schematic drawing of the input sources employed to progress the development of the S-PCG

To evaluate the current standards of care and the needs for improvements, clinical field observations were performed at different units caring for patients with palliative care needs, as well as patient records audits. An overview of S-PCGs development process is shown in Fig. 1.

#### Modelling a new palliative care guide

The S-PCG was designed to include five different elements:

- Guidance on how to identify the patient that may have palliative care needs.
- A systematic approach for a comprehensive assessment of palliative care needs including recommendations for specific validated assessment tools to identify specific symptoms, problems and needs.
- Guidance to negotiate goals of care, care planning and care coordination.
- Symptom and needs-oriented care plans to give concrete, evidence- and experience-based suggestions for personalised evidence-based care activities.
- Guidance for taking care of the deceased and bereavement support.

**Fig. 2:**
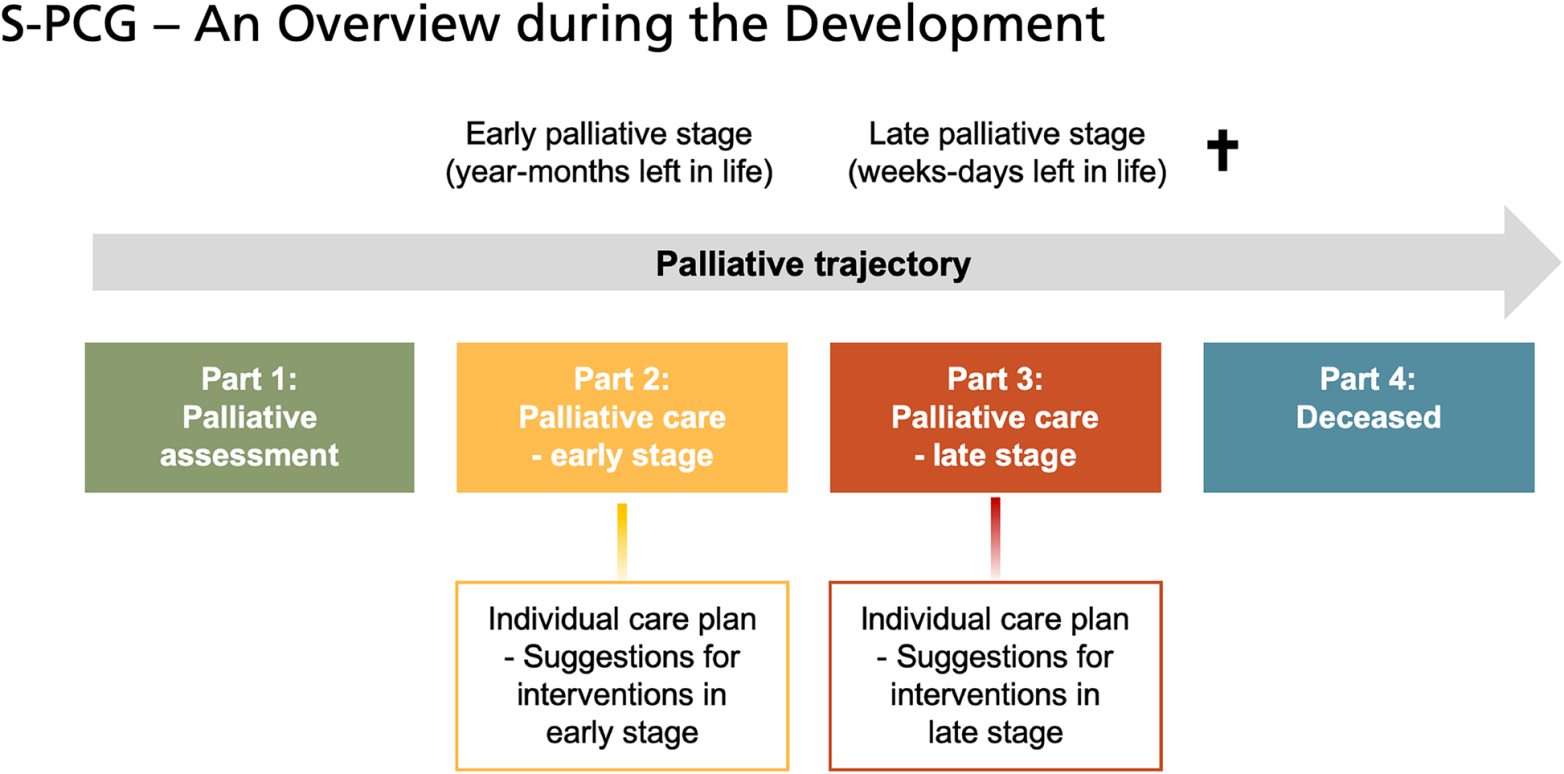
Schematic drawing of the S-PCG documents, during the development of the S-PCG, arranged according to the palliative trajectory. The S-PCG consisted of four parts (six documents) during the development process, that together cover care during the last year of life and promote support to the bereaved family after death of the patient

The first test-version of the S-PCG was drafted by the project group in 2013, initially consisting of four parts covering the palliative trajectory (Fig. 2).

### Patient, family and public involvement

Patient-, family- and public representatives were assigned to the project, to critically review the S-PCG and provide written as well as oral feedback to each version of the S-PCG, during recurrent meetings. Also, to co-create an S-PCG patient information brochure that was produced. To gain more insight into the priorities and wishes of patients and their family, semi-structured interviews were performed with a total of 11 patients and family members, used to shape the content of the S-PCG. Focus-groups interviews were also performed with representatives from two senior organisations. A total of 300 patients were involved during the feasibility testing of the S-PCG.

### Feasibility and pilot testing

In agreement with the MRC framework and as recommended by the Ten Step Implementation Model from the International Collaborative for the Best Care of the Dying Person [54], pilot tests were performed to test the feasibility and usefulness of the newly designed S-PCG. The S-PCG was tested in a total of three pilot tests to ensure that it was feasible to use in broad clinical settings. Since the S-PCG is aimed towards patients with palliative care needs throughout the palliative trajectory, irrespective of diagnosis or the place of care, the only recruitment criteria for the care units were that they were based within the Swedish health care setting and serving adult patients that had or might have palliative care needs. The number of care units, patients and S-PCG documents used in the pilot tests are outlined in Table 1. The number of pilot tests were not pre-decided but constantly evaluated throughout the process. The need for further pilot-testing of the S-PCG was evaluated by the project group and the steering committee after each pilot test.

After each Pilot test period the experiences of staff were evaluated via focus groups interviews, written feedback and review of the documentation in the S-PCG used during each test-period. Feedback was also collected from other health care professionals, patients- and public representatives as well as the national interdisciplinary advisory committee who reviewed the content of the S-PCG parallel to the feasibility testing. An example of the items checked during the evaluation can be seen in Supplementary table B.

The collected feedback, from each pilot period, was then gathered in a large matrix. A thematic analysis inspired by Braun and Clarke [55, 56] was used and the feedback categorized based on: The relevance of the content; usability/user-friendliness; if anything was missing; or redundant; and other comments such as teamwork, implementation needs and patient involvement. The results of the feedback were then thoroughly discussed in the project group and the steering committee and used to improve the next test-version of the S-PCG (Fig. 1).

**Table 1:** Number of patients participating, and S-PCG documents tested in pilot tests I – III

Clinical Test	Number of care units	Number of Patients	Number of S-PCG documents tested						
			**Part 1**	**Part 2**	**Part 2** **care plan**	**Part 3**	**Part 3** **care plan**	**Part 4**	**Total**
Pilot test I	7	28	6	11	-	16	16	13	62
Pilot test II	6	22	13	1	1	6	9^b^	0^c^	30
Pilot test III	34	250	16	62	46	148	144	148	564
**Total number**	**40**^a^	**300**	**35**	**74**	**47**	**170**	**169**	**161**	**656**

^a^Seven of the care units participated in more than one pilot test

^b^More patients were enrolled into the care plan for Part 3 than into the decision support of Part 3 itself

^c^The test units at the hospital had a well-functioning care plan for after the death in their digital hospital records, that prior to the start of the test had been revised to ensure that all of the content from S-PCG part 4 was included

#### Pilot test I

For the first feasibility study, units from different health care services were recruited, including five nursing homes (one of which specialized in dementia care), a specialized palliative home care service, and a general home care service. The selection of care units for Pilot test I, was based on their own initiative, i.e. they contacted us for quality improvement support and showed interest in testing the care plan that was under development.

Prior to initiation of the feasibility study, the personnel (n=166) underwent two days of training and the S-PCG documents together with written tutorial were handed out. The study ran for four consecutive months (May-August 2014).

During the study, 28 patients received care according to the S-PCG. As their diseases progressed, the majority of patients required care according to more than one part of the S-PCG, and thus in total over 60 documents from S-PCG Part 1-4 were used in *Pilot test I* (Table 1).

In addition to the patient and public representatives and the interdisciplinary advisory committee, nurses (n=11) from different specialist care units (i.e. surgery, nephrology, cardiology, haematology, pulmonology and home-based palliative care) also critically reviewed S-PCG Part 2 documents, including the care plan. This was due to the novelty of recommending interventions specifically for patients early in the palliative trajectory.

The collected feedback from *Pilot test I* was compiled, analysed, condensed and then categorised in relation to; the content itself, the usability, functionality and the relevance, and evaluated by the project group. The first test version of S-PCG was considered to be relevant and gave clear and structured support throughout the palliative care trajectory. Apart from comments about the layout and wording, the users requested some adjustments aimed for the care of the elderly. They also raised questions about what was needed for a successful implementation of the S-PCG in the team, such as information and knowledge. The most significant updates after *Pilot test I* included: adaption to better meet the needs of elderly patients with multiple chronic diseases, enhanced focus on the wishes and priorities of the patient, and adaptions to facilitate the working procedures of the team. The updated version of the S-PCG was denoted *test-version 2* (Fig. 1).

#### Pilot test II

Since *Pilot test I* only included care units from municipalities and specialized palliative care there was a need to include units from hospital care in *Pilot test II*. Therefore, the *S-PCG test-version 2* was subsequently tested at a nephrology department at a central hospital and five associated dialysis units in surrounding local hospitals. These units had all taken the initiative to contact us and volunteered to participate in the testing of the care plan. Prior to initiation of *Pilot test II*, a training session was arranged with the personnel (n=90), and an instruction manual was handed out. A designated contact person from each unit received additional training in order to be able to provide on-site support. *Pilot test II* ran for three consecutive months (December 2014-February 2015), after which the experiences of the staff were evaluated as described above. During the second pilot test, 22 patients received care according to the S-PCG (Table 1), with a total of 30 S-PCG documents being used.

In addition to the review from the interdisciplinary advisory committee and the patient and public representatives, semi-structured interviews with patients and next of kin and focus group discussions with representatives from senior organisations were carried out. The patients, their next of kin and the senior public representatives generally considered the S-PCG to be a clear and professional support for the staff, highlighting important issues, and placing their needs in focus.

Additional comments were collected from other professions, that had been underrepresented during the evaluation but highly relevant to the development of the S-PCG. This included assistant nurses, dieticians, occupational- and physiotherapists, municipality care-managers, spiritual representatives, and delegates from the Swedish Registry of Palliative Care.

Furthermore, a content validity test was performed together with five care units that had not participated in *Pilot test I* or *II*: two specialized palliative care-unit, one oncology unit at a hospital, and two geriatric nursing homes. Each unit used the S-PCG for a minimum of 10 patients before giving feedback.

All the collected input from *Pilot test II* together with the content validity test, was compiled and categorized as described before, and used to further develop the S-PCG into *test-version 3* (Fig. 1). The feedback from the content validity test was very similar to the feedback from *Pilot test II*. The results showed that the S-PCG was, for the most parts, easy to understand and fill in — although some found it minorly confusing. Comments were made on a lack of clarity in the layout and in determining when to use the different parts of the material. The S-PCG was considered very comprehensive but at the same time everything was considered relevant. All units whished for the S-PCG to be made available in digital form, within their own patient records system. The most significant updates made to the S-PCG after *Pilot test II* were layout adjustments to give a clearer overall overview of patient needs; adaption to better facilitate cooperation between different users; and the addition of the S-PCG logotype. Further adjustments were also made to the user-manual, clarifying how to use the different parts of the S-PCG.

#### Pilot test III

The *S-PCG test-version 3* was tested between October and December 2015. To ensure variation and broad testing of the S-PCG, *Pilot test III* included 34 care units in various settings within specialized palliative care, municipalities and hospitals.

As before, a training program was provided to the personnel (n=89), particularly to the new units (n=27 units), concerning the structure, content and usage of the S-PCG. Furthermore, selected representatives from the units got in-depth training and were given the task to support the implementation and evaluation processes on site. During *Pilot test III*, 250 patients received care according to the S-PCG with a total of 564 S-PCG documents being used (Table 1).

The evaluation of *Pilot test III* followed the previous described structure. The results highlighted the importance of education, of the managers’ involvement and the need of cooperation and communication, between different professions and different healthcare providers. The content of the *S-PCG test-version 3* was considered useful for the care of an enlarged number of patients, increased the opportunity to discuss the patient’s problems in real time and became a support for the staff’s shared overall view of patient needs and facilitated the planning of the care. However, the content was also perceived as lengthy and, layout was in various need of simplification. Comments, also reflected an overall expressed preference for a digital format. Also, as the focus was more on care needs rather than prognosis, users experienced difficulty in differentiating between early and late phase, i.e. between S-PCG part 2 and 3 (see Fig. 2). Furthermore, unnecessary re-documentation of the same information was also experienced, if shortly after initiation of S-PCG part 2, the patient was identified as dying and needed care according to part 3.

To address this, one of the most substantial changes after the evaluation during *Pilot test III* was merging parts 2 and 3. A circular table of contents was added at the front of each of the three parts to facilitate and clarify that the use of the S-PCG is always based on the patient needs, and specification of support for the children as next-of-kin was moved to an appendix. Due to the extensive number of different digital medical records systems in Sweden it was decided not to provide S-PCG as a digital medical record at this stage of development, but rather encourage thorough imbedding of the S-PCG into the existing medical records already in use.

Supplementary table C gives an example of the general feedback provided during *Pilot test III*, together with some of the main changes made to the S-PCG.

## Results

**Fig. 3:**
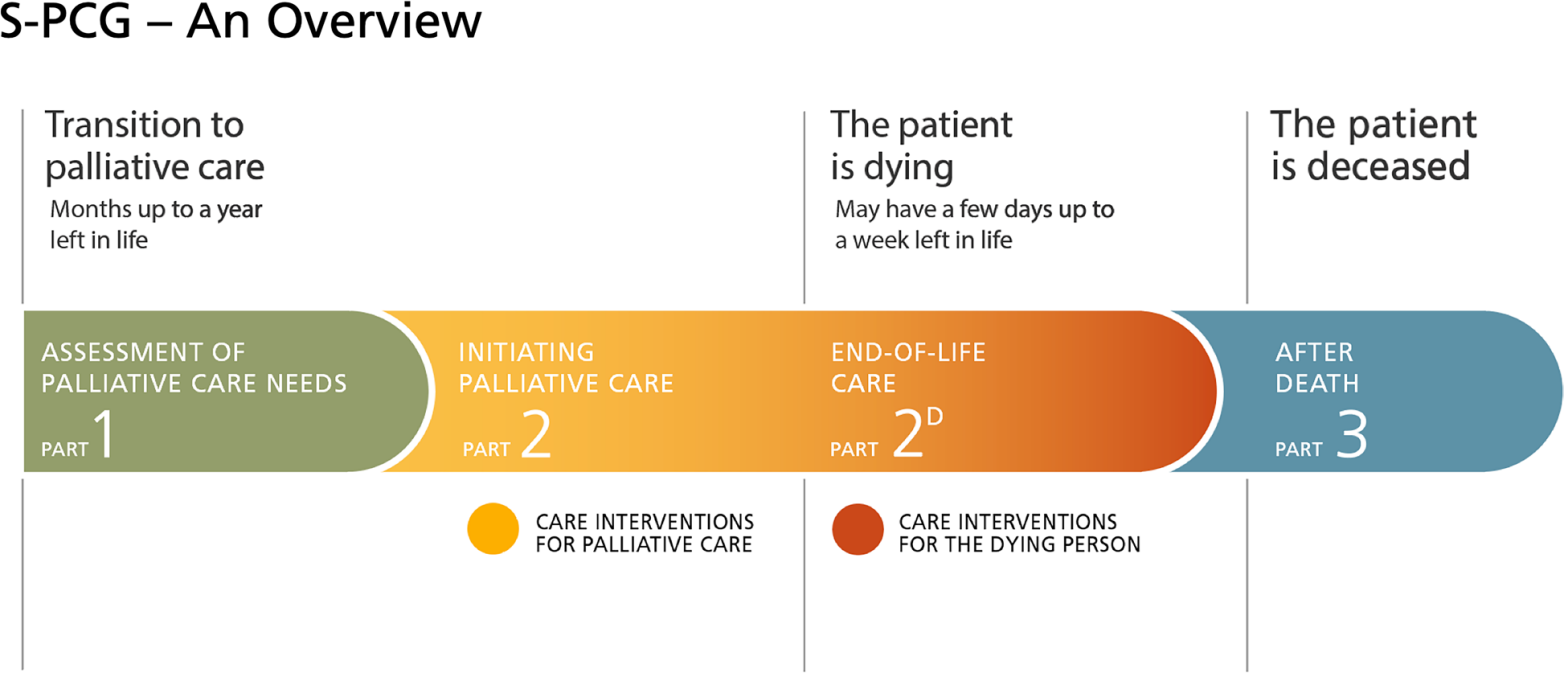
Schematic drawing of the S-PCG documents Version 1.0 (at the time they were launched), arranged according to the palliative trajectory. The S-PCG Version 1.0 consists of three parts (six documents) that together cover the care during the last year of life and support to the bereaved family after death of the patient

This article outlines the development of a novel *Swedish palliative care guide* (S-PCG) intending to improve the end of life care for adult patients irrespective of diagnosis. The extensive and expansive stepwise multi-unit and interprofessional testing and evaluation procedures resulted in *S-PCG Version 1.0*, which was launched in September 2016. It consists of three parts and includes, in total, six documents (Fig. 3). The S-PCG version 1.0 was reviewed and assessed by the International Collaborative for the Best Care of the Dying Person, which stated that the S-PCG was an excellent care plan, detailed and comprehensive. It was approved by the International Collaborative to be congruent with the principles and core elements for the best care for the dying person [54].

The S-PCG was designed to give support and structure to health care professionals when meeting adult patients with potential palliative care needs, irrespective of diagnosis. The S-PCG can be initiated at any stage of the palliative trajectory by choosing the relevant part that is best suited to meet the current needs of the patient. It provides a structure to identify the patients’ status and needs through assessing symptoms, function, social situation as well as to highlight the importance of capturing the patient priorities and wishes. We promote the use of validated assessment instruments such as the Integrated Palliative Outcome Scale (IPOS) [57, 58], Edmonton Symptoms Assessment Scale (ESAS) [59] and the Abbey Pain Scale [60] within the S-PCG. By capturing the individual care-needs, the S-PCG can help create an overall picture and shared understanding of the needs and possibilities beneficial for each patient’s quality of life and of his next of kin. Table 2 gives an overview of the main topics and sections that are included in the S-PCG.

**Table 2:** Overview of S-PCGs key topics, sections and example of the issues included in the S-PCG

Key topic	Section/item	Example of issues or tools
**SYMPTOM AND STATUS**	Symptoms and status	Assessment of symptoms and status with validated tools such as IPOS^a^, Abbey Pain scale or ESAS
	Communication skills	The patient’s ability to communicate or need for assistance (e.g. interpreter)
	Function in daily life	Assessment of level of function (ECOG^c^) and activities of daily living (ADL)
**COMMUNICATION/ DECISIONS**	End-of-life conversation	Regarding prognosis and focus of care; Treatment interventions and life-sustaining treatments preferences
	Medical decisions	Regarding current medical interventions, treatments and DNR^d^; Prescription for anticipatory medication
	Information	Practical information for the patient and/or the family (e.g. brochures, available benefits, support groups)
	Understanding	Insight about current prognosis and focus of care
**PREFERENCES**	Wishes and priorities	What is important right now; Spiritual and cultural needs; Involvement in care and treatment
**SOCIAL CONTEXT**	Family^e^	Family members distress/worries; Involvement in care; and Need for support
	Children	Minor children in the family and assessment of their need for information and support
**PLANNING**	Coordination of care	Contact information and need for referral (e.g. to specialized palliative care, dietician, religious/spiritual leader)
	Care interventions	Individual care interventions together with suggestions of possible interventions for each symptom/condition
	Reassessment and consent	Plan for new assessment of palliative care needs; Consent to share information with other care providers
**LAST DAYS OF LIFE**	Signs of dying	Signs that the patient might be dying (e.g. the patient is bedridden; deteriorating level of consciousness)
	Recognition of dying	Recognition by the physician that the patient may be entering the last days of life
	Special requests/needs	Special requests and needs of the patient and/or family before and/or after the death (e.g. rituals, symbols)
	Care of the dying	Continuous assessment of symptoms and status, and care interventions during the last days of life
**AFTER THE DEATH**	Care of the deceased	Practical, spiritual and cultural procedures and routines after death
	Bereavement support	Information to the family (e.g. about practical issues, grief and support groups) and bereavement support

^a^IPOS = Integrated Palliative care Outcome Scale (58*). ^b^ESAS = The Edmonton Symptom Assessment System (59*). ^c^ECOG = The Eastern Cooperative Oncology Group performance status

^d^DNR = Do-not-resuscitate order. ^e^The concept family is used here in its broadest sense and includes all persons of significance to the patient. * Refers to the reference-number in the reference list

Table 2. Overview of the key topics and sections in the S-PCG, with examples of issues/tools included in the care guide.

### The different parts of S-PCG

S-PCG Part 1 is a two-page concise tool that provides simplified support for the identification of the patients’ palliative care needs and initiate care planning. It can be used wherever patients with palliative care needs are encountered, for example in general practice, nursing homes and in- and outpatient hospital care. It can be used during consultations or as an assessment tool for multi-professional team rounds.

S-PCG Part 2 is an in-depth assessment of the same topics as in the Part 1, and is intended to support the provision of care for patients with palliative care needs regardless of time left in life. Part 2 consists of a guideline for initiating palliative care, assessment tools, and an associated care plan for recommended interventions for common symptoms and problems, which can be individually initiated according to the identified care needs of the patient. It focuses on defining common goals for care, and may support decisions and palliative care in the time range of months or up to a year left in life. Part 2 also has an appendix regarding children as next of kin.

S-PCG Part 2^D^ can be initiated when a patient is assessed as likely dying. Part 2^D^ adds on to Part 2, but focuses on the issues and symptoms that are frequent in the last few days of life. It includes guidance to recognise the dying phase in and hence initiate discussions on shifting the goals of care in the awareness of a most likely soon approaching death. Dying patients require frequent attention and symptom assessment, which is now thoroughly supported, including frequent reassessment in the care plan of Part 2^D^.

S-PCG Part 3 comprises a clear and condensed guide and thorough plan for care after death, in accordance with Swedish national care standards [50, 61, 62]. It supports relevant routines after a patient has died, including recommendations on how to care for the deceased person and promotes bereavement support for the family, including children in the family.

#### Implementation and dissemination of the S-PCG

The development of the S-PCG started as a local initiative based on national recommendations. It has been well received by regional and national palliative care authorities and organisations and has been given support by the Swedish National Board of Health and Welfare. It is now included in the Swedish National Palliative Care Guidelines [63].

Lessons were learned from the Liverpool Care Pathway (LCP) [28] which was phased out in 2014 as a consequence of a critical governmental report entitled “More Care, Less Pathway” [64]. This statement and the possible risk that guidelines develop into checklists, supported our effort in operationalizing not only knowledge but also the palliative care approach into the novel care guide.

To facilitate a robust implementation, all parts of the S-PCG, information and support materials are openly available at the website of The Institute for Palliative Care [65]. The documents for clinical use are accessible after registration. The managers of the registered units are responsible for the local application of the S-PCG including securing staff training and quality monitoring. Regular follow-up of results from the Swedish Registry of Palliative Care as well as audits of patient records are recommended. An audit tool has been designed to assist this procedure.

Brochures and instructional films of the S-PCG have been made available online [65]. An educational program has been developed for units aiming to implement the S-PCG and, to make it accessible to more users, an online S-PCG educational program is under development. Theme days/workshops for registered S-PCG units, aimed for education, inspiration and networking have also been arranged and the S-PCG has been presented at several conferences both in Sweden and internationally.

The S-PCG has been well received by the health care personal and at the beginning of the year 2021 a total of 305 care units were registered as S-PCG users in Sweden. Some regions have made the use of S-PCG compulsory within their district. The S-PCG has now been incorporated within several digital patient record systems in Sweden and research programs evaluating the clinical impact and effect of S-PCG in different care settings have been initiated.

## Discussion

We have now developed a care guide (S-PCG) that helps to identify adult patients with palliative care needs early and right through to end-of-life. It provides assessment tools and structured plans for documentation and guidance to support continued personalized palliative care. We have described the initial development of S-PCG, aimed to provide a link between evidence based best-practice care according to the core principles of palliative care, and professional behavior in everyday clinical practice.

Methodological challenges included the processing of the extensive information and feedback from the various care settings, health care professionals as well as patients and families. However, the collected expertise of the participants is unique and has contributed substantially to the development of S-PCG throughout the palliative care trajectory.

A majority of those who gave feedback on the S-PCG during the final pilot testing confirmed that the content was relevant to a broad group of patients and gave a good overall understanding of patients’ needs. It was perceived as a good support to clinical practice, although it is worth mentioning that the majority of the participating care units contacted us expressing a need for a care guide and on their own initiative volunteered to participate in the testing of the S-PCG. This might predispose respondents to a more positive attitude towards the care guide, thus affecting the result of how the guide was received. However, it can also be noted that in many of the testing units the decision was made by the managers and not all personnel that gave feedback were positive towards the S-PCG from the beginning.

Although the majority of the users were positive towards the use of S-PCG, it was at the same time seen as very comprehensive, time consuming and it was confusing to the users when to use the different parts of the S-PCG. It is essential to routinise screening for palliative care needs within clinical practice [22] and for that to happen it is important not only to pilot test the instrument during the developmental stage but also to take into account the users’ feedback into the final product. Based on the feedback, we made some significant changes to the design of the S-PCG, such as merging part 2 and 3 to make the documentation more efficient and user-friendly, without compromising the content. We also clarified the instructions on when to use the different parts of the S-PCG and emphasized a thorough planning of the use before implementation.

The S-PCG includes a brief guidance to screen for patients with potential palliative care needs. Apart from the “surprise question” regarding prognosis [66], the items covering disease stage, functional decline, disease progression and symptom burden are formulated to be fully transparent to the patient and family. The widely used surprise question gives a prognostic perspective, can be used as a reflective tool for team members, and together with other tools such as the PCST (palliative care screening tool) may help clinicians to identify patients with palliative care needs [67, 68]. As our intention was not to use a scoring system but rather to merely support the clinical assessment, the surprise question was not included in the main S-PCG documents as a criterion for potential palliative care needs. Instead it was highlighted in the user manual.

To promote transparency, we made it a priority for the content of the S-PCG to be understandable and non-offensive to patients and family members who may want to read these documents. We therefore included patient-, family- and public representatives in the discussion of the content of the S-PCG. In the planning and execution of the next MRC phases [48] (evaluating the implementation and the use of the S-PCG) we will intensify our partnered work with patients and families – strengthening user involvement from the level of consultation, to eventually, reach collaboration and equal partnership [69].

The potential limitation of not performing our own systematic review of the relevant scientific literature is, in fact, overshadowed by our access to ongoing updates in national recommendations and relevant evidence-based documents that were used [9, 13, 50, 70]. Further, the large group of health care professionals and patient representatives ensured clinical experience and gave relevant guidance when other sources did not contribute the substantial knowledge than one could wish for.

The strengths of the S-PCG is that regardless of medical diagnosis and whether the patient is being treated in a hospital, at home, or in a nursing home or hospice, the S-PCG can provide structure and guidance for the care. It puts the patients’ needs in focus and is designed to promote communication between different caregivers and encourage collaboration between health care professionals and the patient and their family. However more research is needed and the S-PSG will be updated continually based on new scientific evidence as well as clinical experience, the users input and patients and their families experience.

## Conclusions

After extensive development work and broad testing, the S-PCG has the potential to provide meaningful support in identifying palliative care needs; facilitates inter-professional assessment and care of these patients; and emphasizes the needs of the family throughout the palliative trajectory. It supports high-quality personalised palliative care, and when properly used may help patient and families express their too-often-neglected needs, support individual negotiation of goals of care, and subsequently promote relevant care. Choosing to implement S-PCG includes responsibility for its use in concordance with the principles of good palliative care. The next step entails scientific evaluation of the clinical impact and effect of S-PCG in different care settings – including implementation, patient and family outcomes, and experiences of patient, family and staff.

## Supplementary Information

**Additional file 1.**

**Additional file 2.**

**Additional file 3.**

## Data Availability

The datasets generated and/or analysed during the current study are not publicly available due to legal restriction as described by the Swedish law regarding data of sensitive nature and data protection, but can be available from the Institute for Palliative Care in Lund, Sweden on reasonable request.

## Abbreviations

**S-PCG**: The Swedish Palliative Care Guide
**MRC**: The Medical Research Council
** LCP **: Liverpool Care Pathway
**IPOS**: Integrated Palliative Outcome Scale
**ESAS**: Edmonton Symptoms Assessment Scale
