# Supplementary material for: A novel care guide for personalised palliative care – a national initiative for improved quality of care
Source: BMC Palliat Care. 2021 Nov 11;20:176. doi: 10.1186/s12904-021-00874-4 (PMC8582140; doi:10.1186/s12904-021-00874-4)
Supplement: Supplementary file 1 — Additional file 1. [file 12904_2021_874_MOESM1_ESM.docx]

**Supplementary table A.** Professions represented in the steering committee, project group and in the interdisciplinary advisory committee.

|  | **N** | **Areas of responsibility/speciality** |
| --- | --- | --- |
| **Steering committee** | **5** | Professor in palliative medicine (n=1), professor in palliative care nursing (n=1), associate professor in hematology (n=1), associate professor in palliative care nursing (n=1), project manager and quality improvement nurse (n=1) |
| **Project group** | **10** | Palliative care nursing (n=4), quality improvement nursing (n=2), palliative care research (n=2), palliative medicine (n=1), physiotherapy and public health (n=1) |
| **Interdisciplinary advisory committee**  **Professions:** | **95** |  |
| Registered nurses | 41 | Palliative care (n=18), community nursing (n=11), oncology (n=4), cardiology (n=2), surgery (n=2), geriatric (n=1), lung (n=1), neurology (n=1), dementia care (n=1) |
| Innovation improvement leads | 12 | Palliative care (n=6), oncology (n=2), surgery (n=1), geriatric (n=1), community care (n=1), hospital care (n=1) |
| Physicians | 11 | Palliative medicine (n=7),  oncology (n=2), cardiology (n=1), GP (n=1) |
| Assistant nurses | 8 | Palliative care (n=3), community care (n=3), oncology (n=1), geriatric/neurology (n=1), |
| Community matrons | 5 | Community care (n=5) |
| Unit managers | 5 | Community care (n=3), palliative care (n=1), geriatric (n=1) |
| Dieticians | 3 | Palliative care (n=2), geriatric (n=1) |
| Occupational therapists | 2 | Palliative care (n=1), quality improvements within occupational therapy (n=1) |
| Social workers | 2 | Palliative care (n=2) |
| The Swedish Register of Palliative Care personnel | 2 | National quality register (n=2) |
| Researchers | 2 | Palliative care (n=2) |
| Physiotherapist | 2 | Palliative care (n=2) |
