# Supplementary material for: A novel care guide for personalised palliative care – a national initiative for improved quality of care
Source: BMC Palliat Care. 2021 Nov 11;20:176. doi: 10.1186/s12904-021-00874-4 (PMC8582140; doi:10.1186/s12904-021-00874-4)
Supplement: Supplementary file 2 — Additional file 2. [file 12904_2021_874_MOESM2_ESM.docx]

**Supplementary table B:** Example of items checked to evaluate the feasibility and usefulness of the S-PCG, during the pilot testing.

| **Sources and methods for collection of information** | **Themes/items checked** | **Clarification** |
| --- | --- | --- |
| **CLINICAL TESTS (PILOT I-III)**  Focus groups interviews with clinical users  Written feedback from clinical users  Content validity test, through focus groups interviews and a questionnaire  **INTERDISCIPLINARY ADVISORY COMMITTEE**  Written feedback, on each part of the S- PCG, section by section  Whole day workshop^1^ with the group | **Relevance of the content** | Is the content relevant and adequate for the care of people with palliative care needs and for your workplace?  Is the content of the S-PCG easy/difficult to understand and use? |
|  | **Usefulness / User-friendliness** | Can you describe your experience of participating in the testing of the S-PCG? What has been good and what needs to change? Can you describe what it has been like to work with each part of the S-PCG? Advantages and disadvantages of the S-PCG? |
|  | **Missing issues** | Is there anything missing from the S-PCG documents? Anything that you would like us to add to the S-PCG? |
|  | **Redundancy** | Is the S-PCG too detailed? Is there anything that you think is unnecessary to include in the S-PCG and could be removed? |
|  | **Teamwork – Communication** | How has the cooperation within the team during the test of the S-PCG? Have you been able to involve all team members? What has worked well and what has not worked well? Has the S-PCG test influenced or initiated discussions about your current palliative care practices? If so, what discussions have you had? |
|  | **Patient- and family**^2^ **involvement** | Do you think the S-PCG has added any value for patients and their family? Can you describe how it has been to involve patients and families in the S-PCG? In what way did you involve patients and families? If not, what prevented you from doing so? How do the patient, family and public representatives perceive the concepts and the vocabulary of the S-PCG?  Was there anything in the S-PCG that felt inappropriate or offensive to the patient and family? |
|  | **Implementation** | What training/information do you think is needed for the introduction/start of the S-PCG? Or the implementation of S-PCG? What do you think about the S-PCG user manual? Is it usable? Is it too comprehensive or is it missing some information? Do you think you could implement the S-PCG at your workplace with the information given? |
| **PATIENT-, FAMILY- AND PUBLIC REPRESENTATIVES**  Focus-groups meetings  Written feedback on each part of the S-PCG, section by section | **Perception of the S-PCG based on the themes mentioned above** | What do you think of the S-PCG in general? Is it adequate? Is the content important? Is there anything that you think is important that is missing from the S-PCG? Is there anything that you think is unnecessary to include in the S-PCG and should be removed. |
|  | **Perception of concepts and vocabulary used in the S-PCG documents** | How do you perceive the concepts and the vocabulary of the S-PCG? Was there anything that was difficult to understand? Was there anything in the S-PCG that felt inappropriate or offensive? Is there anything that you think should be changed/rephrased/removed? |
| Semi-structured interviews with patients that were admitted to the S-PCG during Pilot II and their family^2^ member | **Perceptions about the different topics in the S-PCG, and if and what complementary written information is needed** | Would you like to tell us what you think about us asking about:  Symptoms?; How daily life works for you?; If you have had an informative conversation with your doctor about your diagnose and prognose?; Your understanding of the situation with your disease and the prognose?; Your wishes and preferences for the care?; Your social network and what you/your family needs help with?; If there are children in the family^2^ and about their needs for support?; The possible need for other health care contacts (e.g. dietician, counsellor, district nurse)?; Your consent for the relevant staff to access the information in this material?; Your wishes and preferences regarding your participation in decisions about care and treatment?  Do you think that this topic is relevant/ important to you? or do you think it should not be included in the S-PCG? Do any of the issues we´ve discussed today felt inappropriate or offensive? Is there anything else that you think is important for health care professionals to ask for or attend to that we have not discussed? Is there any information that you think is important to get in writing, e.g. in a brochure? |
| **REVIEW OF THE PATIENT RECORDS USED IN THE PILOT TESTS I - III** | **How the S-PCG documents were used during the pilot tests** | We reviewed: The number of S-PCG documents included; number of patients allocated to each part of the S-PCG; if the question was answered/the assessment was made*;* any obvious misunderstandings in using S-PCG; what items had been added that possibly should be included in S-PCG; what care-interventions had been used and added; which questions had not been answered; which profession documented each question; if there had been a team collaboration. |

**1)** Workshop with the interdisciplinary advisory committee was only held once, during Pilot test II. **2)** The concept family is used here in its broadest sense and includes all persons of significance to the patient.
