# Supplementary material for: A novel care guide for personalised palliative care – a national initiative for improved quality of care
Source: BMC Palliat Care. 2021 Nov 11;20:176. doi: 10.1186/s12904-021-00874-4 (PMC8582140; doi:10.1186/s12904-021-00874-4)
Supplement: Supplementary file 3 — Additional file 3. [file 12904_2021_874_MOESM3_ESM.docx]

**Supplementary table C:** Summary of the general feedback^1^ from the evaluation of the S-PCG during Pilot test III, with an example of comments from the clinical test users; patient-, family^2^- and public representatives; the interdisciplinary advisory committee and the issues addressed/ raised after the evaluation.

| **Items checked** | **Comments from the clinical test users** | **Patient-, family**^2^**- and public representatives** | **The interdisciplinary advisory committee** | **Summary of issues addressed/raised after the evaluation** |
| --- | --- | --- | --- | --- |
| **RELEVANCE OF THE CONTENT** | **The content is relevant**  "S-PCG has a good content, all the important elements are included"  “S-PCG is suitable for all our patients”  “Relevant content and we almost didn’t need to add anything”  **Gives a good overall picture of the patient needs**  “You get the “overall look of things” with S-PCG”  “S-PCG provides a good overview, gives consensus and encourages holistic approach for the whole person”  **Is S-PCG suitable for all care forms?**  "Should there be different versions for different types of care?"  “There is no time to have those long conversations in the hospitals”  “Most of our patients (in the nursing home) do not have minor children, that section takes up a lot of space” | **Important content**  “S-PCG is a good document that addresses important issues”  “Good, it is important to ask the family for their need for support”  “It is detailed but I think detailed questions are better than big overall ones” | **Relevant content**  “Good and clear content”  “Yes, the content is relevant and I think it is good that you have the care activities listed as well, you can choose those that fit the patients’ needs”  **Is the same document suitable for all care forms?**  “Would it maybe be better with different documents…for example, one for home care and one for hospital care” | **The content**  The content of the S-PCG did not change much, small adjustments were made in word phrasing.  A decision was made to hold on to one version of the S-PCG, suitable for all care-settings. This, was done because the fundamental concept of the S-PCG is centered around the individual patient needs. This was highlighted in the creation of a user’s-manual, an educational program and in S-PCG brochures.  Only the basic questions for children as family^2^ were kept within the main documents and the more specific support was moved to an appendix, to be use when needed. |
| **USEFULNESS –**  **USER-FRIENDLINESS** | **S-PCG gather all information and is easy to use**  “It was an advantage that all information is gathered in one place”  “Easy to use – since not everything has to be filled in for every patient”  “It is good that S-PCG first find the problem and then guide us to take action to solve/alleviate it”  **Very comprehensive and time consuming**  “Time-consuming”  “Very comprehensive”  "Comprehensive material but good that you can choose what is relevant for each patient" | **Feels clear and professional**  “S-PCG feels professional”  “S-PCG is clear/ straightforward”  “Nothing in the S-PCG feels inappropriate or offensive”  “Didn't think concepts and wording were inappropriate or needed to be changed, it seemed professional”  “Some words are maybe more medical terms but this is also aimed for the professionals and that feels safe” | **Clear and usable**  “Yes, the documents are clear and seem to be usable”  “S-PCG part 3 and part 4 are integrating well in my Palliative Care Unit”  “The overview paper in the Part 2 care measurements is very helpful”  **Too comprehensive and time consuming**  “A standing reflection is that the S-PCG documents are too comprehensive and take a very long time to complete” | **Changes of the structure and layout**  A circular table of content was added to clarify that the use of the S-PCG does not require documentation in the order they are presented and that it is always based on the patient needs. To highlight this, checkboxes for “not applicable at this moment” were also added to several sections of the S-PCG documents. |
| **Items checked** | **Comments from the clinical test users** | **Patient-, family**^2^**- and public representatives** | **The interdisciplinary advisory committee** | **Summary of issues addressed/raised after the evaluation** |
| **USEFULNESS –**  **USER-FRIENDLINESS**  **(continued)** | **S-PCG is a good support for the care of the patient**  “S-PCG puts palliative care in focus and clarifies what you can do for the patient”  “S-PCG gives good support to new staff and new graduates so they will know what to do”  “Exceptional support to have during conversations with the patient and the family”  **Difficult to decide when to use part 2 and 3 of the S-PCG**  “The care plan for part 3 is good and shows clearly what we are doing for the patient, but is starts too late”  “If a patient is admitted to S-PCG Part 2 and then shortly after he is dying and needs to move onto part 3, then we need to fill in all the same information again, that is a lot of work”  “Same information in Part 2 and part 3, feels like a repetition” |  | **Keep the support-questions visible**  “I think that the support questions for the patient wishes and priorities should be visible in the S-PCG document. These are good questions” | **Changes of the structure and layout (continued)**  The support-questions, for the patient wishes and priorities, were moved from the appendix into the main document.  Parts 2 and 3, of the S-PCG, were merged together, into Part 2 and 2D (D for the dying phase), to address the problem with repetition and double documentation. The titles of part 2 and former part 3 (now 2D) were changed to give a clearer description.  Also, the aim and a short description of when to use each specific part was added to the front page of each part of the S-PCG. |
| **MISSING ISSUES** | **Clearer instructions on how to use the S-PCG**  “It should be made clearer that you can skip items that are not relevant for the patient you are caring for”  “Better instructions before the use, I am not sure how to use the different parts of the S-PCG”    **“Clearer layout and more space for text”**  “More space for the answer’s text/comments”  “Can you expand the comment field under wishes and priorities”  “Better structure for the care interventions in part 3”  **S-PCG in digital form**  “S-PCG in digital form so you can type in the computer and then print it out for the patient records”  “The S-PCG needs to be included in the digital patient records system” | **Ask about pets or something else that can be worrying for the patient**  “They need to ask if there is something else I am worried about, such as who is taking care of my cat”  “Information about who is responsible for what” | **More space for ADL and care interventions for edema**  “More space for writing what ADL-aids the patient has” | **Layout and support for implementation**  Clearer layout was created, with more space for text as suggested. The instructions on how to use the S-PCG were clarified in the user-manual.  **Patients whishes**  A question about pets was added to the “support questions” under the section about patient’s wishes and priorities.  **S-PCG in digital form**  Due to the extensive number of different digital medical records systems in Sweden it was decided not to provide S-PCG as a digital medical record at this stage of the development, but rather encourage thorough imbedding of the S-PCG into the existing medical records. Digital interactive PDF documents of the S-PCG were however created for download. |

| **Items checked** | **Comments from the clinical test users** | **Patient-, family**^2^**- and public representatives** | **The interdisciplinary advisory committee** | **Summary of issues addressed/raised after the evaluation** |
| --- | --- | --- | --- | --- |
| **REDUNDANCY** | **Nothing is redundant**  “Nothing, S-PCG is comprehensive but everything is important”  “Nothing is redundant”  **Double documentation**  “It would be double documentation to have status updates and a medication list in the S-PCG since it is in our digital patient records already”  “Risk for double documentation”  “Part 4 is good but we already have a good checklist for after the death in the computer. No need for both” | **Nothing needs to be removed**  “Everything in the material is essential and nothing needs to be added or removed."  “Can’t think of anything that you can remove from the care guide” | **Everything can be relevant at some point**  “Nothing, everything can be relevant to some patient at some point”  **Other comments**  “You need to sign very often in the documents, in different places. Is that necessary?” | **Not much could be removed**  Overall very few could point out anything to remove from the S-PCG, so apart from small changes in paraphrasing, the content stayed the same.  The medication list was removed from the S-PCG and an opportunity to refer to the digital medication list was added to the documents. |
| **TEAMWORK – COMMUNICATION** | **S-PCG leads to discussion within the team about important issues**  “S-PCG is a well drafted document that leads to good discussions on the important issues”  “Good that S-PCG makes the end-of-life conversations visible to everyone in the team”  “The S-PCG has started discussions about our current way of working”  **Resistance in the beginning and difficulties to get all team members on board**  “Difficult to get employees on board in the beginning but it got better later”  “Difficult to get doctors on board”  **S-PCG can highlight the teamwork**  “S-PCG can have a positive impact on the teamwork”  “As a doctor I can say that S-PCG lands more on the nurses’ side, but it is not overwhelming and there is much gained once part 2 has been done”  “Testing the S-PCG confirms that we (in our team) have very good procedures for the care”  “The S-PCG makes the assistant nurses feel more responsible for, and more involved in the palliative care”  “S-PCG clarifies what the assistant nurses do in the team – GOOD!” | **Give patients and family**^2^ **a clear information about who to contact and where to get support**  “Contact information, we meet so many caretakers and I don’t know who I can call”  “The health care must help the patient to know where to go, who to talk to, or refer to others if the patient's needs cannot be met.”  “The personnel need to make sure that the relatives are guided towards the right person/appropriate support when needed” | **S-PCG demands teamwork**  “S-PCG demands teamwork which is good” | **Highlighting the importance of teamwork**  The online film about the S-PCG and what to think about before implementation includes topics about the importance of teamwork and tips on how to include the team in the implementation of the S-PCG.  **Guide the patient towards those that are needed**  Space for written information about who to contact and contact information was added to a patient and family brochure. |

| **Items checked** | **Comments from the clinical test users** | **Patient-, family**^2^**- and public representatives** | **The interdisciplinary advisory committee** | **Summary of issues addressed/raised after the evaluation** |
| --- | --- | --- | --- | --- |
| **PATIENT- AND FAMILY**^2^ **INVOLVEMENT** | **Patient involvement was not at barrier**  “Information about S-PCG to patients and relatives is not perceived as a barrier”  **Family felt reassured and the support was improved**  “S-PCG provides reassurance to the family members, once they have been informed about the care plan”  “Before the family were asking questions such as: has he/she gotten any pain-medicine recently? But now they can just see what we have done in the S-PCG, and all of a sudden there is time for other type of conversations”  **S-PCG clarifies the palliative care needs and the work that is being done**  “S-PCG clarifies the palliative care needs”  “The family sees what we work with in the S-PCG and feel that it is important what we do”  “It is good to go through the S-PCG, you know what the patient and the family want”  **Some experienced barriers to involve the family**  “It can be difficult with the needs of the family, they can have many needs and we want to focus our time on the patient” | **Creates security for the family**  “S-PCG creates security for the family, you check if the document is filled in” |  | **Patient involvement**  A brochure with information for patients and their family was created together with patient- and family representatives.  The importance of patient involvement was raised in an online film about S-PCG users-experience as well as in the S-PCG education program. |
| **IMPLEMENTATION** | **Information and training in the S-PCG is needed**  “More training is needed on S-PCG”  “You need to go through it together in the team to feel comfortable before you start using the S-PCG”  “Important to have knowledge of the S-PCG if it is to work”  “The S-PCG is good and useful but you need training in order to understand how to use the documents”  **Education in palliative care is needed**  “With a lot of new staff, education in basic palliative care is needed”  “Doctors need more knowledge of palliative care in the hospitals”  “Training in basic palliative care for everyone” | **Highlight the importance of having a conversation with the family without the patient around**  “To put in the user guide that it is important to sometimes have conversation with the family members without the patient around. When you ask questions about how the family member is holding up or if he/she needs support it is sometimes difficult to answer honestly if the patient is close by.” |  | **Brochures**  A brochure containing short introduction on what S-PCG is and how it is intended to be used was created and made available both in print and online.  Another brochure was created with recommendations before implementation highlighting the importance of good planning before implementation.  The third brochure includes short practical users-instructions, aimed to be carried in the clinical work if needed.  The fourth brochure is aimed for patients and their families. |

| **Items checked** | **Comments from the clinical test users** | **Patient-, family**^2^**- and public representatives** | **The interdisciplinary advisory committee** | **Summary of issues addressed/raised after the evaluation** |
| --- | --- | --- | --- | --- |
| **IMPLEMENTATION**  **(continued)** | **Time to plan the use and the documentation-routines**  **of the S-PCG**  “Time for planning the implementation”  “To go through and decide on how the documentation procedures and routines should be, before the use is important”  “We missed more time for planning”  **Support and understanding from managers**  “More involvement of the manager”  “More support and assistance to the engage staff”  “The nurses wanted to test the S-PCG on all of the patients but the management said no” | **Include patients’ stories in the education**  ”I suggest that you record stories from patients with the question ‘What is important to me? ’ and include it in the training/introduction of NVP to healthcare professionals. So, they don’t forget why they are doing this work”  **Provide guidance and support for the health care personal to perform end-of life conversations.**  “Before implementing S-PCG on a new ward each clinic should ensure that they involve someone within the practice who is experienced in dealing with the difficult conversations, such as telling someone that they are dying. So that this person can guide and be supportive to the others” |  | **Four films were created**  A film about the S-PCG including, among other things, interviews with patient, experts in palliative care, managers and health-care personnel as well as staged material.  Another film aimed to inspire managers and leaders.  A third film about how to use and what to think about before implementing the S-PCG.  A forth film about the users experience of using the S-PCG in clinical practice.  All films are openly accessible online (65)^3^.  **Education**  The need for more training and preparation before implementation, as well as clearer instructions on how to use the S-PCG, was highlighted in the user-manual, in our films and in our brochures. A whole day educational program about S-PCG was also created, including, among other things, stories from a patient that was filmed.  To address the need for education in basic palliative care, links to on-line education in palliative care were provided. |

**1)** The more detailed feedback that was given for each section of every part of the S-PCG was very comprehensive and is not included in the table. This could include comments such as “*Move section 2.8 further ahead*” or “Under section 3.10, change: *contact with religious leader* to *contact with religious-/spiritual leader*” or “*add a checkbox for not applicable under section 4.5*”. All comments were however taken into consideration and discussed within the project group during the evaluation of the S-PCG.

**2)** The concept family is used here in its broadest sense and includes all persons of significance to the patient.

**3)** See reference number 65 in the reference list.
